# Fatigue in Chronic Respiratory Diseases: Theoretical Framework and Implications For Real-Life Performance and Rehabilitation

**DOI:** 10.3389/fphys.2018.01285

**Published:** 2018-09-19

**Authors:** Mathieu Gruet

**Affiliations:** Université de Toulon, LAMHESS, Toulon, France

**Keywords:** performance fatigability, perceived fatigability, muscle function, exercise training, ecological momentary assessment, chronic obstructive pulmonary disease, cystic fibrosis, obstructive sleep apnea

## Abstract

Fatigue is a primary disabling symptom in chronic respiratory diseases (CRD) with major clinical implications. However, fatigue is not yet sufficiently explored and is still poorly understood in CRD, making this symptom underdiagnosed and undertreated in these populations. Fatigue is a dynamic phenomenon, particularly in such evolving diseases punctuated by acute events which can, alone or in combination, modulate the degree of fatigue experienced by the patients. This review supports a comprehensive inter-disciplinary approach of CRD-related fatigue and emphasizes the need to consider both its performance and perceived components. Most studies in CRD evaluated perceived fatigue as a trait characteristic using multidimensional scales, providing precious information about its prevalence and clinical impact. However, these scales are not adapted to understand the complex dynamics of fatigue in real-life settings and should be augmented with ecological assessment of fatigue. The state level of fatigue must also be considered during physical tasks as severe fatigue can emerge rapidly during exercise. CRD patients exhibit alterations in both peripheral and central nervous systems and these abnormalities can be exacerbated during exercise. Laboratory tests are necessary to provide mechanistic insights into how and why fatigue develops during exercise in CRD. A better knowledge of the neurophysiological mechanisms underlying perceived and performance fatigability and their influence on real-life performance will enable the development of new individualized countermeasures. This review aims first to shed light on the terminology of fatigue and then critically considers the contemporary models of fatigue and their relevance in the particular context of CRD. This article then briefly reports the prevalence and clinical consequences of fatigue in CRD and discusses the strengths and weaknesses of various fatigue scales. This review also provides several arguments to select the ideal test of performance fatigability in CRD and to translate the mechanistic laboratory findings into the clinical practice and real-world performance. Finally, this article discusses the dose-response relationship to training and the feasibility and validity of using the fatigue produced during exercise training sessions in CRD to optimize exercise training efficiency. Methodological concerns, examples of applications in selected diseases and avenues for future research are also provided.

## Introduction

Fatigue is an important debilitating symptom which concerns virtually all chronic respiratory diseases (CRD). Fatigue is a leading cause of consultations in CRD with major clinical implications. Despite its well-acknowledged negative consequences on patient's life, fatigue is still a misunderstood and underdiagnosed symptom in CRD. As a consequence, there is currently no intervention that has been developed specifically to treat all aspects of this ambiguously defined symptom in CRD. Fatigue is rather often considered as a secondary outcome in interventions aiming primarily to increase physical fitness and/or health-related quality of life.

Previous research on fatigue in CRD has been based on many dichotomies and no consensus as yet emerged on how to define and measure this symptom in these specific populations. The present review adopts a terminology of fatigue adapted from Enoka and Duchateau (2016) that includes the subjective sensations of fatigue (i.e., perceived fatigability) and the objectives changes in performance (i.e. performance fatigability), both being closely interrelated and inseparable. Most studies in CRD evaluated perceived fatigability as a trait characteristic. Using multidimensional scales, such studies provided important insights on the prevalence and the clinical consequences of fatigue in these populations (e.g., Stridsman et al., 2015; Nap-Van der Vlist et al., 2018). However, these scales do not permit to capture and understand fatigue over time and across contexts and environments. Evaluating fatigue as a state is thus fundamental to shed light on the dynamics of fatigue experienced by CRD patients in real-world settings.

Fatigue as a state can be evaluated both at rest at a specific moment of the day (e.g., before bedtime, after lunch) and during a physical activity. Surprisingly, only very few studies evaluated fatigue as a state at rest in CRD and there are no specific validated tools available for that purpose in these populations. During an ongoing physical activity, the fatigue experienced by a patient will be determined by the rates of changes in both perceived and performance fatigability. Various tests have been used to evaluate performance fatigability in CRD. Performance fatigability depends on the ability of the peripheral muscles and the central nervous system to meet the demands of the prescribed task. Both systems can exhibit abnormal changes in response to exercise in CRD (e.g., Maltais et al., 2014; Marillier et al., 2018a) and contribute to increased performance fatigability. Thus, a performance fatigability test should allow an easy implementation of measures of both peripheral and central contributors of fatigue. However, with the aim to be used in clinical trials and even routine practice, the test should also demonstrate satisfactory feasibility and reliability. Importantly, mechanistic studies on the neurophysiology of CRD-related fatigue often display poor external and ecological validity. This is notably the case for local fatiguing exercises (e.g., Marillier et al., 2018a). It is often unknown whether the results from a performance fatigability study could be generalized to circumstances beyond the actual research setting and how such results could be translated to real-life performance and rehabilitation. Can the results of a specific fatiguing task in a given subgroup of CRD patients be extrapolated to another task in patients with different disease severity? And, at least as important, can the same results be relevant in the real daily-life? The real challenge of a performance fatigability test is to cumulate all the aforementioned characteristics to be suitable in most CRD patients in both clinical and research settings.

A better knowledge of the determinants of fatigue is a prerequisite to develop new strategies aiming to reduce the influence of each underlying factor in the daily life of the patients. However, such knowledge may also paradoxically enable to produce more fatigue in the specific context of exercise training. Following the principle of muscle loading, seminal reports in chronic obstructive pulmonary disease (COPD) (e.g., Burtin et al., 2012) demonstrate that quantifying the production of fatigue following an acute exercise training session was an effective strategy to identify future responders and non-responders to an exercise program. The key issue is then to understand why some CRD patients are not able to exhibit significant fatigue following a given exercise training session and then to propose individualized countermeasures.

This review aims first to clarify the terminology of fatigue and considers the strengths and weaknesses of the current models of fatigue and their applicability in CRD. This article then briefly reviews the prevalence and impact of fatigue in CRD and considers the advantages and drawbacks of the scales intending to assess fatigue as a trait and as a state in these patients. This review then proposes a step-by-step approach to identify the ideal test of performance fatigability in CRD and to translate the mechanistic laboratory findings into the clinical practice and real-life performance. Finally, this article discusses the viability of using the fatigue produced during an acute exercise training session in CRD to detect the future responders to an exercise program. The present review is enriched by several practical examples of application in various CRD and provides directions for future research.

## Traditional dichotomies, limitations of current knowledge, and new models of fatigue

Many works on fatigue, in both clinical and sports research areas, have been based on traditional dichotomies. The usual way is to consider *mental, psychological, cognitive, perceived* fatigue distinctly from *physical, physiological, muscle* fatigue or fatigability. It is also common to adopt a *peripheral* vs. *central* (*spinal* vs. *supraspinal*) dichotomy to suggest a locus of the observed *muscle* fatigue (e.g., Gruet et al., 2013). Other distinctions based on temporality or methodologies are also usually made (e.g., *chronic* vs. *acute* fatigue, fatigue as a *trait* vs. fatigue as a *state, subjective* vs. *objective* fatigue) (Enoka and Duchateau, 2016). The word “fatigue” is thus most of the time preceded by one of the aforementioned adjectives, leading to as many different definitions as adjectives. Although each definition may be relevant in isolation, such compartmentalization does not favor the emergence of a comprehensive interdisciplinary approach of fatigue and rather confines the study of fatigue to a monodisciplinary research. Each discipline has developed its own corpus of knowledge, taxonomy, experimental models, and methodologies. For instance, neurophysiologists often consider a neuromuscular approach of fatigue. *Muscle* fatigue is then defined as a reduction in maximal force of power generated voluntary by a muscle or a muscle group and/or a reduction in twitch force elicited by nerve stimulation (Gandevia, 2001). This condition is reversible by rest and should be differentiated from muscle weakness which can be defined as an impaired capacity to generate force (NHLBI Workshop summary, 1990). Study of muscle fatigue is relevant in CRD as many patients present a peripheral muscle dysfunction (e.g., muscle atrophy, inflammation, metabolic abnormalities; Maltais et al., 2014; Gruet et al., 2017) and even sometimes cerebral abnormalities (e.g., gray matter decrease; Esser et al., 2016). A neuromuscular approach of fatigue, giving consideration to both central (i.e., corticospinal drive) and peripheral (i.e., peripheral muscle transmission and contractility) factors is thus relevant in these patients to understand the mechanisms underlying increased fatigability during a physical task. However, such approach is clearly irrelevant to capture the fatigue experienced by patients at “rest,” independent of any ongoing physical activity. On the other hand, perceived fatigue experienced at rest and captured by questionnaires is not sufficient to understand how and why fatigue progressively develops during a given physical activity of daily living (e.g., walk up several flights of stairs). Thus, a multidimensional approach of fatigue is clearly warranted, especially in multi-systemic and progressive diseases that are CRD, with the aim to understand the fatigue experienced by patients in various contexts and at different times during the course of the disease.

Spruit et al. (2017) recently proposed a model of fatigue in patients with COPD. Moderate to severe fatigue can be perpetuated by various factors, grouped into three categories: systemic factors (e.g., cardiovascular disease, exercise-induced oxidative stress), physical and psychological factors (e.g., breathlessness, symptoms of anxiety and/or depression) and behavioral (e.g., nocturnal awakening, low social support). Fatigue can be precipitated by infectious COPD exacerbation and its treatment. This model suggests that the fatigue experienced by these patients is not simply the consequence of the COPD disease and cannot be predicted by the sole degree of airflow obstruction. Fatigue is rather the consequence of multiple factors that may act alone or in interaction, at rest and during/after a physical exercise. If some factors such as physical activity levels and exacerbation rate have been demonstrated to play a significant role in perpetuating and precipitating fatigue in COPD (Baghai-Ravary et al., 2009; Andersson et al., 2015), the influence of some other factors still has to be demonstrated. It is of note that this model mixes, at the same level, factors at a macro- (e.g., physical deconditioning, physical inactivity, cardiovascular disease) with factors at a more micro-level (e.g., systemic inflammation, breathlessness), the latter being often dependant on the former. This approach also does not make a clear distinction between factors influencing perceived and performance fatigue and thus does not rely on a unified taxonomy of fatigue. This model may thus be challenging to be experimentally tested but is so far the most comprehensive model of fatigue in COPD.

Contemporary models of fatigue have criticized the traditional peripheral/central dichotomy and rather suggested that fatigue is the result of a complex interaction between physiological activity and psychological state. For instance, in their Integrative Governor theory, St Clair Gibson et al. (2018) proposed that the competitive, dynamic interplay between physiological and psychological homeostatic drives regulates exercise performance and the fatigue process. In the same vein, Enoka and Duchateau (2016) proposed a definition of fatigue that includes (1) the “perceived” component, referring to the sensations about fatigue, and (2) the “performance” component, referring to the capacity of the neuromuscular system to meet the requirements of a given task. Importantly, the definition also suggests the interdependence relationship of the two components. The authors defined fatigue as “a disabling symptom in which physical and cognitive function is limited by interactions between performance fatigability and perceived fatigability.” The term “fatigability” is used here to normalize the level of fatigue experienced by a person relative to the requirements of a given task that produces it. Based on this taxonomy, the same authors proposed a generic model of fatigue (adapted and modified from Kluger et al. (2013), initially developed for neurological diseases). In this model, perceived fatigability depends on two domains: maintenance of homeostasis (e.g., blood glucose) and psychological state (e.g., mood). Performance fatigability also depends on two domains: contractile function (e.g., calcium kinetics) and muscle activation (e.g., activation patterns). This model has the advantage of being universal and can be virtually adapted for every disease including CRD, the relative weight of each factor and their reciprocal interaction depending on the disease.

**Figure 1:**
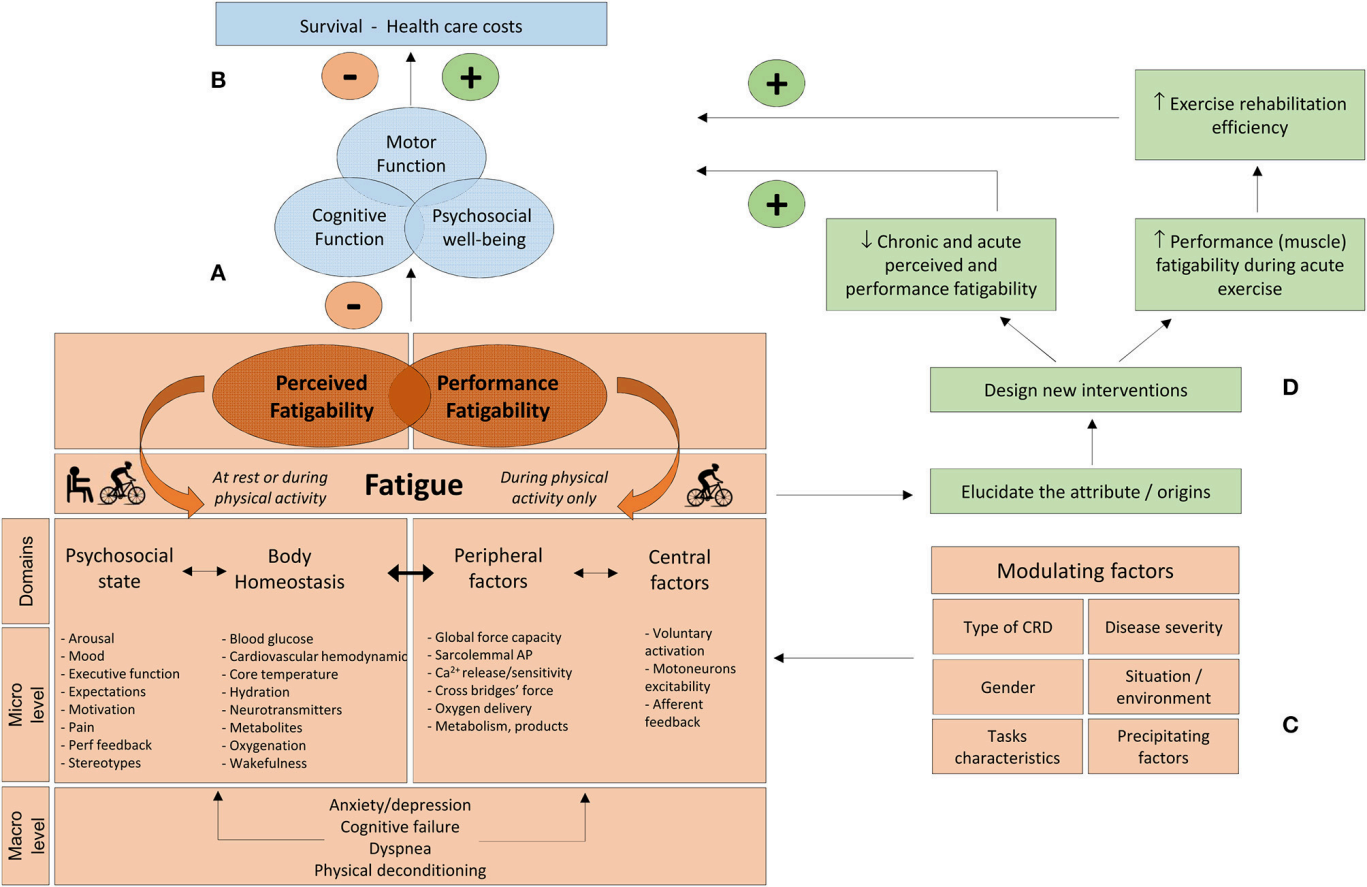
**(A)** Performance and perceived fatigability concept is adapted and modified from Enoka and Duchateau (2016). The domains of perceived and performance fatigability are controlled by different factors acting at a micro- and macro-level. **(B)** Consequences of fatigue on patient's life. **(C)** Modulating factors which can influence the respective weight of each factor contributing to fatigue. **(D)** A better knowledge of the determinants of fatigue will permit to design new individualized strategies with the aim to increase acute muscle loading during a given exercise training session and counteract the negative influences of fatigue (see text for details). CRD, chronic respiratory diseases; Perf feedback, performance feedback; sarcolemmal AP, Sarcolemmal action potential.

The model proposed in the present review and adapted for CRD is based on this taxonomy (Figure 1A). The designation of the domains of perceived and performance fatigability was slightly modified. “Psychological state” was replaced by “psychosocial state” to underline the possible influence of coping with social identity threats on fatigue. Indeed, copying with negative stereotypes (which can be prevalent among CRD, e.g., Johnson et al., 2007) may increase fatigue (Inzlicht and Kang, 2010; Chalabaev et al., 2013). The factors of perceived fatigability are those described by Enoka and Duchateau (2016), with the addition of “stereotypes” and “cardiovascular hemodynamic” (which can also impact fatigue levels, e.g., blood pressure, cardiac output, stroke volume; Freeman and Komaroff, 1997; Nelesen et al., 2008). “Central factors” and “peripheral factors” were chosen to designate domains of performance fatigability. Four macro-factors which are particularly prevalent in CRD (i.e., dyspnea, anxiety/depression, cognitive failure, and physical deconditioning) can exert an important influence on several of the micro-factors underlying domains of perceived and performance fatigability (Figure 1A). The present model also mentions the potential modulating factors and consequences of fatigue and suggests a step-by-step approach to develop specific countermeasures. The different parts and components of this model will be developed throughout the subsequent sections.

Perceived and performance fatigability can both exert a negative influence on motor, cognitive, and psychosocial functioning, all of which, in isolation and/or combination, may in turn compromise survival and increase medical care costs (Figure 1B). However, various modulating factors must be considered when investigating fatigue, each potentially influencing the relative contribution of perceived and performance fatigability underlying factors. Thus, elucidating the origins of fatigue is context-specific and the etiology of fatigue must be considered according to patients (i.e., type of disease, disease severity, gender), tasks (i.e., nature, duration and intensity of the tasks) and situation/environment characteristics (e.g., rest vs. exercise; work vs. home) and precipitating factors (i.e., hospitalization and associated treatments) (Figure 1C). For instance, “oxygenation” and “metabolites” are probably important attributes in severe COPD during exercise. Early activation of the anaerobic metabolism during exercise can result in elevated lactic acid production, precipitating exercise-induced hyperventilation and dyspnea (Maltais et al., 1998), which in turn can contribute to increased perceived fatigability. However, these factors have probably a minor influence at rest in patients with mild cystic fibrosis (CF). Each of the modulating factors presented in Figure 1C can be tested experimentally (e.g., specific inclusion criteria, stratification for disease severity, gender, pre vs. during vs. post hospitalization).

Shedding light on the attributes of fatigue will permit to create new individualized interventions, aiming to (1) increase acute muscle loading during a given exercise session (a prerequisite for training efficiency, see section Fatiguers as Responders: Experimental Evidences and Exercise Training Strategies) and, (2) reduce both chronic and acute perceived and performance fatigability under various conditions. Achieving both of these goals can in turn improve patients' health and thus reduce medical care costs (Figure 1D). This model clearly suggests that fatigue should be evaluated in different ways to reflect the diversity of contexts and environments in which fatigue can emerge over the natural history of the disease. Fatigue can be assessed in four different ways. Perceived fatigability can be evaluated as a trait characteristic (section Fatigue as a Trait: Useful Information on Prevalence and Impact), but also as a state, in the present moment, both at rest and during ongoing physical activity (section Fatigue as a State: Time for Ecological Momentary Assessment). Performance fatigability is evaluated during physical activity (section Fatigability and Performance: Theoretical Concepts and Applications and Performance Fatigability: Which Test With Which Fundamental Characteristics?). Table 1 summarizes four selected methods to evaluate perceived and performance fatigability in CRD. These methods will be discussed in details in the next sections.

**Table 1:** Summary of four selected methods to assess perceived and performance fatigability in CRD.

**Fatigue attribute**	**Trait/State**	**Capture condition**	**methods/tests**	**Main applications/advantages**	**Disadvantages**	**Main future directions**
Perceived fatigability	Trait	At rest	Combination of multidimensional generic scale and disease-specific scale	Elucidate predictors of fatigue. Obtain information about prevalence and clinical consequences of elevated levels of perceived fatigability.	Limited ecological validity. Recall bias. Not optimal for multiple repeated assessments.	Validate disease-specific fatigue scales. Conduct observational and interventional longitudinal studies.
	State	At rest	ROF scale	Capture changes in fatigue over time in various contexts and environments. Impact of specific events and their temporality on perceived fatigability. Ecological validity, feasibility.	Limited details on the attributes of fatigue. Absence of normal values. Long-term compliance.	Assess diurnal and seasonal changes in fatigue. Asses fatigue pre-post acute treatment (e.g., physiotherapy session) and throughout hospitalization for exacerbation or over the course of a new long-term treatment.
	State	During physical activity	ROF scale	Assess the kinetics of perceived fatigability severity during a given physical task. Obtain insight into the global level of fatigue produced by an exercise training session.	Absence of mechanistic insights into fatigue development.	Assess fatigue kinetics during standardized physical activities of daily living (e.g., stair-climbing). Usefulness in combination with markers of muscle fatigue to identify responders to exercise training.
Performance fatigability	State	During physical activity	Intermittent isometric graded contractions	Obtain insight into the neuromuscular factors limiting performance and contributing to elevated state of fatigue during a given physical task.	Ecological validity, feasibility	Assess supraspinal mechanisms in fatigue-related force loss by implementing brain investigations techniques. Assess performance fatigability with concomitant cognitive tasks. Quantify the association between performance fatigability and real-world performance (e.g., walking tests). Determine the effectiveness of specific interventions in reducing performance fatigability.

*For each method is given the main applications and advantages, disadvantages and some directions regarding the future use of these methods. ROF, rating-of-fatigue*.

## Perceived fatigability

### Fatigue as a trait: useful information on prevalence and impact

The trait level of fatigue refers to the amount of fatigue experienced by patients over a preceding period of time, usually several days or few weeks. It is evaluated with multidimensional scales, which constitute by far the most common way to assess fatigue in studies involving CRD patients. These scales reveal that the prevalence of fatigue is elevated in CRD, including COPD (Stridsman et al., 2013), CF (Nap-Van der Vlist et al., 2018), bronchiectasis (Hester et al., 2012), obstructive sleep apnea (OSA) (Mills et al., 2008), lung cancer (Graves et al., 2007), chronic pulmonary aspergillosis (Al-Shair et al., 2016b), sarcoidosis (Bosse-Henck et al., 2017), and idiopathic pulmonary fibrosis (Atkins et al., 2016). These multidimensional scales also provide evidence that fatigue in CRD is distinguishable from other related symptoms also prevalent in these populations, such as sleepiness, dyspnea, anxiety, and depression (Baghai-Ravary et al., 2009; Jackson et al., 2011; Al-Shair et al., 2016a; Atkins et al., 2016; Nap-Van der Vlist et al., 2018). Elevated trait level of fatigue has major clinical implications. Fatigue is associated with reduced quality of life, increased rates of hospitalization, reduced physical activity levels, and exercise intolerance (Baghai-Ravary et al., 2009; Paddison et al., 2013; Andersson et al., 2015; Al-Shair et al., 2016a; Nap-Van der Vlist et al., 2018). Fatigue has also been identified as a predictor of mortality in COPD (Stridsman et al., 2015). No less importantly, fatigue is only partially explained by disease severity and symptoms related to shortness of breath. For instance, apnea severity accounted for only a very small percentage of variance in fatigue score in patients with OSA, whereas factors such as inflammation and depression symptoms were found to be important independent predictors (Bardwell et al., 2003; Mills et al., 2008). Strategies aiming to treat breathing impairments may thus not be sufficient to reduce fatigue and other factors affecting body homeostasis and psychosocial state may be important to consider.

However, methodological considerations should also be taken into account, especially when comparing studies and the clinical implications of these findings. The first reason is related to the variety of scales used to investigate the trait level of fatigue. Hjollund et al. (2007) identified 156 multi-symptom scales (from 670 studies) and 71 scales (from 416 studies) which have been used to specifically measure perceived fatigability, irrespective of the disease. The number of scales used in CRD is certainly much lower but remains important. For instance, Antoniu and Ungureanu (2015) identified 8 multidimensional scales which are commonly used to assess perceived fatigability in COPD. Table 2 summarizes the unidimensional, multidimensional and specific-disease scales which are frequently used in CRD (*n* = 16). Such diversity precludes accurate comparisons between studies as the scores produced by the scales are, most of the time, not interchangeable, as indicated by poor to moderate correlations between scales in previous reports (Vasconcelos et al., 2006; Panitz et al., 2015). It should also be mentioned that CRD are progressive diseases and some questionnaire items may turn inappropriate with disease progression. This should be considered when selecting a fatigue questionnaire, especially for long-term longitudinal studies.

**Table 2:** Scales commonly used to assess perceived fatigability in CRD patients.

**Name of the scale**	**Example of study**	**Example of population**
Borg VAS scale	Al-Shair et al., 2011	COPD
Single question, Likert scale	Chervin, 2000	OSA
Fatigue Severity Scale (FSS)	Ozalp et al., 2012	Bronchiectasis
Short Form Health Survey 36 (SF-36), vitality domain	Antoniu et al., 2016	COPD
Functional Assessment of Chronic Illness Therapy - Fatigue scale (FACIT-F)	Andersson et al., 2015	COPD
Multidimensional Fatigue Inventory (MFI)	Orava et al., 2018	CF
Profile of Mood States (POMS), fatigue subscale	Jackson et al., 2011	OSA
Brief Fatigue Inventory (BFI)	Chen et al., 2018	COPD
Checklist Individual Strength-20 (CIS-20)	Nap-Van der Vlist et al., 2018	CF
Fatigue Assessment Scale (FAS)	Lingner et al., 2018	Sarcoidosis
Identity-Consequences Fatigue Scale (ICFS)	Paddison et al., 2013	COPD
Chalder Fatigue Scale	Jarad et al., 2012	CF
Multidimensional Assessment of Fatigue (MAF)	Belza et al., 2001	COPD
Fatigue Impact Scale (FIS)	Hester et al., 2012	Bronchiectasis
Manchester COPD Fatigue Scale (MCFS)	Al-Shair et al., 2016a	COPD
COPD and Asthma Fatigue Scale (CAFS)	Miravitlles et al., 2013	COPD

*The current list is not intended to be all-inclusive. The light gray indicates unidimensional scales. The intermediate gray indicates multidimensional generic scales. The dark gray indicates specific-disease scales. CF, cystic fibrosis; COPD, chronic obstructive pulmonary disease; OSA, obstructive sleep apnea*.

There is also still a debate whether the trait level of fatigue should be evaluated from generic or disease-specific scales. On one hand, fatigue can be considered as an unspecific symptom as a whole, thus not requiring the development of specific scales for each disease (Hjollund et al., 2007). The use of generic scales may thus allow a better comparison of this symptom between diseases. On the other hand, fatigue can still have distinct features depending on the disease, making a disease-specific assessment important. For instance, pain can be an important correlate of fatigue in rheumatoid disorders whereas it may not specifically be the case in CRD, the opposite being certainly true for dyspnea. Some factors may also play a greater role in causing or maintaining fatigue in a given CRD compared to the other (e.g., sleep-related breathing disorders in OSA). Considering these elements, assessment of perceived fatigability as a trait should ideally be performed by using both disease-specific and generic fatigue scales. Admittedly, the use of two questionnaires may not always be feasible in daily clinical practice. Nonetheless, the few additional minutes to complete a second questionnaire are most certainly worthwhile, at least for research purposes. To date, fully validated CRD-specific fatigue scales are available for COPD and asthma (Al-Shair et al., 2009; Revicki et al., 2010) and further studies should develop and validate disease-specific scales in CRD for which fatigue is an important issue.

The vast majority of studies investigating the trait level of fatigue in CRD used cross-sectional designs. In most studies, the scores of fatigue are linked to the scores of other symptoms and some clinical data (e.g., Stridsman et al., 2013; Atkins et al., 2016; Nap-Van der Vlist et al., 2018). Such designs have the advantage to demonstrate excellent feasibility, resulting in the completion of large studies across the disease spectrum, even in rare CRD (e.g., sarcoidosis, Bosse-Henck et al., 2017). However, such studies have the disadvantage to assess fatigue only at a single time point and thus may not precisely reflect the level of fatigue experienced by the patient over the course of his long-lasting disease. Moreover, the cross-sectional association between two parameters does not permit to infer on the causes and consequences. For instance, previous reports demonstrated a link between physical activity levels and fatigue scores across numerous CRD, including COPD (Andersson et al., 2015) and CF (Nap-Van der Vlist et al., 2018). Lack of physical activity can be a mechanism through which fatigue occurs (notably via peripheral muscle deconditioning and reduced force capacity) but an elevated trait of fatigue can also prevent some patients to engage in regular physical activities. Longitudinal observational and interventional studies, controlling for confounding factors are clearly warranted to obtain further insights on the factors underlying elevated trait of fatigue and on its development and evolution over the natural history of the disease. Some recent clinical trials demonstrated the effectiveness of multidisciplinary pulmonary rehabilitation programs to reduce perceived fatigability. For instance, both a 3- and 12-week program have proven effective in reducing fatigue, in patients with sarcoidosis (Lingner et al., 2018) and COPD (Peters et al., 2017), respectively. However, in both studies fatigue was chosen as a secondary outcome and it is not known which component of these multimodal programs actually reduced fatigue. Importantly, multidimensional scales may not be well-suited for longitudinal studies, especially when conducting repeated assessment of fatigue over a short-time period. Other scales are thus necessary to conduct repeated measurements of perceived fatigability over a short period of time, for instance to capture diurnal changes in fatigue or throughout hospitalization for exacerbation.

### Fatigue as a state: time for ecological momentary assessment

As discussed above, measuring the trait level of fatigue of CRD patients is essential to shed light on the prevalence, the etiology and the clinical consequences of fatigue as well as to assess its changes in response to therapeutic interventions. However, retrospective self-reports of fatigue, as measured by multidimensional scales during clinical routine or research visits are subject to recall bias and are not well-adapted to capture changes in fatigue over time in various contexts and environments. Fatigue assessment at a specific moment in time refers to the state level of fatigue.

Ecological momentary assessment (EMA) is a range of methods collecting real-time data on individuals' current behaviors and experiences in real-world situations (Stone and Shiffman, 1994). This method is an alternative to retrospective reports which fail to capture the dynamics of patients' everyday life, on both long-term (e.g., month by month) and short term basis (e.g., day-to-day, hour by hour). EMA methodology seems particularly adapted to capture the state level of fatigue in CRD and that for several reasons. First, perceived fatigability varies with context and environment and thus should be captured during real-life situations. Moreover, CRD are progressive diseases punctuated by several particular events which can exert, alone or in combination, substantial changes in fatigue. Fatigue is not a stable symptom over time and can fluctuate in an unpredictable manner within and between days. In healthy subjects, the state level of fatigue measured at rest during several time points (7 times from 10-min after waking until bedtime) augments linearly throughout the day whereas the daily average amount of fatigue augments linearly throughout the working week (Monday to Friday) and decreases to reach its minimal values during the weekend (Micklewright et al., 2017). This scenario may not apply in CRD patients, as the state level of fatigue may be modulated by several factors within and between days (e.g., diurnal variation in mucus accumulation, schedule and sequence of the treatment, adherence to the treatment, occurrence of an exacerbation).

Treatment regimens have considerably evolved over the years to manage CRD, with the development of complex strategies which require increasing time and effort. For instance, many children and adults with CF spend more than 2 h/day on respiratory therapy (i.e., including nebulized, oral, airway clearance and exercise therapies as well as maintenance of materials), even exceeding 30 h/week in some patients (Sawicki et al., 2009; Hafen et al., 2013). However, increased treatment burden is associated with poor adherence, which is common among CRD patients and associated with many clinical issues (e.g., increased rates of morbidity, hospitalizations, health care costs, reduced quality of life) (Bourbeau and Bartlett, 2008; Bishay and Sawicki, 2016).

According to the working-capacity model of Heckman et al. (2015), the fatigue related to treatment can be modulated by four different factors: 1- increased general demands (i.e., daily-life tasks) and 2- increased treatment burden (i.e., amount of effort required for a given treatment), which define, in combination, “patient demands”; and 3- reduced general resources (i.e., psychosocial and cognitive functions) and 4- illness burden (i.e., disease symptoms), which define, in combination, “patient capacity.” The balance between patient demands and capacity will modulate the fatigue related to treatment which in turns determines patient adherence. For instance, when patient capacity decreases for a given demand, treatment fatigue will increase and patient adherence will decrease. Perceived fatigability is determined by rates of changes in body homeostasis and psychosocial state (see traditional dichotomies, limitations of current knowledge, and new models of fatigue and Figure 1). Now let's consider the following scenario. A treatment may reduce perceived fatigability through improvements in body homeostasis. For instance, continuous positive airway pressure treatment improves some attributes of body homeostasis (e.g., oxygenation, blood pressure, sleepiness) in patients with OSA (Antic et al., 2011; Gottlieb et al., 2014). However, in the long-term, the treatment loses some effectiveness whereas the psychosocial state is affected (for instance because alterations in some of its attributes, e.g., motivation, mood, expectations). The positive changes in body homeostasis do not compensate enough for alterations in psychosocial state, leading to increased perceived fatigability. In the model of Heckman et al. (2015), it translates to increased treatment fatigue because patient demands exceed coping capacity, eventually resulting in decreased treatment adherence. Within this framework, the repeated assessment of the state level of fatigue throughout the duration of a treatment may help in predicting future changes in health behavior. Small changes in fatigue may indicate the need to reconsider the ratio between demands and capacity, with the aim to redress the balance. This can be achieved for instance by reconsidering patients' preference for a given treatment especially when several strategies displaying only small differences in term of effectiveness are available. This is for instance the case of some contemporary airway clearance therapies (Bott et al., 2009; Flume et al., 2009). Thus, other factors than effectiveness should guide the individual's choice of techniques. Increasing patient's satisfaction may reduce treatment burden, improve psychosocial state, reduce perceived fatigability and in turn promote sustained changes in health behavior (e.g., long-term adherence).

Repeated EMA of fatigue may also have interest during an exacerbation-related hospital admission. For instance it would be beneficial to determine the suitability of exercise training during severe exacerbations to reduce the state level of fatigue at discharge, both at rest and during activities of daily-living. Long-term EMA assessment (i.e., month-by-month) of fatigue should also be conducted to determine whether fatigue displays seasonal variation in CRD. It can be speculated that higher state levels of fatigue are encountered during winter, mirroring the seasonal variations of pulmonary exacerbations and/or hospital admissions and/or acquisitions of respiratory pathogens (Psoter et al., 2013, 2017; Donaldson and Wedzicha, 2014; Williams et al., 2017). The knowledge of a potential seasonality of fatigue in CRD may increase our understanding of the mechanisms underlying elevated perceived fatigability in these populations and may guide the development of prevention strategies.

The fundamental question is now to determine which scale should be used for EMA of fatigue in CRD. Despite all the aforementioned potential applications, the state level of fatigue has only been scarcely investigated in CRD and not in the context of EMA. Instead, the state level of fatigue has been evaluated in specific acute situations. For instance, two studies (Al-Shair et al., 2009, 2016a) used Borg's ratings of perceived exertion (RPE) scale (Borg, 1982) before and after a six-minute walk test (6MWT) as a measure of a state level of fatigue. However, perceived exertion should be distinguished from perceptions of fatigue. Perceived exertion refers to the subjective experience of how heavy/laborious it feels to work/to exercise and should be viewed as an indicator of physical strain (Borg, 1982; Micklewright et al., 2017). Perceived fatigability refers to the sensation of reduced ability to cope with mental and physical stressors in relation to a given task and environment (Micklewright et al., 2017). As a consequence, perceived exertion can only be experienced during acute episodes of physical exercise, and RPE scores should immediately drop to zero right after exercise cessation. On the opposite, perceived fatigability can be evaluated both at rest, during and after an exercise (with a gradual decrease during post-exercise recovery). Such assumptions are supported by the high correlation between RPE and fatigue scores during graded cycling exercise, which disappears during a 30-min recovery period (Micklewright et al., 2017).

In view of these elements and of the absence of specific validated fatigue scales which can be used in various contexts, environments and populations, Micklewright et al. (2017) developed and validated the rating-of-fatigue (ROF) scale. The ROF scale consists of 11 numerical points, ranging from 0 (“not fatigued at all”) to 10 (“total fatigue and exhaustion—nothing left”), with 5 descriptors and 5 pictures. This scale demonstrated satisfactory face validity and high levels of convergent validity during graded cycling exercise to exhaustion, resting recovery and activities of daily living. The ROF should thus be perfectly adapted for repeated EMA in all the above-described situations in CRD. An important next step will be to validate the ROF scale in the specific context of CRD and then to implement this scale in dedicated software in order to benefit from the multiples advantages of using mobiles technologies for EMA. Some available software may be customized to the needs of a specific study (e.g., capturing ROF scores at a precise time of the day). The advantages of using mobile-EMA platforms over paper-and pencils methods for EMA are discussed elsewhere (Shiffman et al., 2008; Wen et al., 2017). Briefly, it may reduce non-compliant behaviors such as backfilling and thus augment the ecological validity of the data. Mobile-EMA platforms can also ascertain the timeliness of patients' responses and thus provide an objective measure of response compliance. Pending the development/customization of software to implement specific validated fatigue scales, existing EMA dedicated software can be used to collect responses to various questions, including patients' fatigue. This is notably the case of a large multicenter ongoing study investigating the trait and the state level of fatigue in patients with COPD and its associations with exacerbation-related hospitalizations and mortality (see study protocol in Goertz et al., 2018). This is the first study using EMA to capture fatigue in COPD. This study uses an EMA application (i.e., PsyMate^TM^, https://www.psymate.eu/, installed on an iPod) allowing to record answers about fatigue, context and surroundings, at eight random moments of the day, for 5 consecutive days at baseline and then at 4, 8 and 12 months.

Fatigue as a state can also be measured during a physical task. Capturing and understanding the state level of fatigue during physical tasks is essential for at least two reasons: first, physical activities represent an important part in the everyday occupations. Second, a greater modulation of the attributes of perceived and performance fatigability is expected during physical tasks and severe fatigue can emerge rapidly in these situations. The level of fatigue experienced by a patient during a physical task will be determined by the rates of changes in body homeostasis and psychosocial state, but will also be modulated by alterations in peripheral muscle function and the ability to voluntary activate the involved muscles (i.e., central activation) (see Figure 1 and traditional dichotomies, limitations of current knowledge, and new models of fatigue).

As a symptom, the intensity of fatigue that develops during a given physical task can be captured by specific scales. Again, ROF scores should be differentiated from RPE scores, although they correlate during graded exercise (Micklewright et al., 2017). During whole-body exercise such as running or cycling, it is possible to report perceived exertion as differentiated “feelings,” with the difficulties of breathing (i.e., dyspnea) reported separately from the perceived effort in the active limb muscles (Bolgar et al., 2010). For instance, some reports have demonstrated that many patients with CF, despite their important ventilatory limitation, had subjective symptoms of muscle effort in excess of symptoms of dyspnea during submaximal and maximal cycling exercise (Moorcroft et al., 2005; Gruet et al., 2010a, 2018; Quon et al., 2015). These observations provide indirect, subjective evidences of an important implication of the peripheral muscles in the exercise intolerance experienced by these patients. It is also possible, beyond measuring the intensity of dyspnea with a RPE scale (e.g., 0–10 Borg Scale) to report qualitative dimensions of dyspnea during graded exercise. For instance, Quon et al. (2016) demonstrated that the onset of “unsatisfied inspiration” occurred at a lower relative exercise intensity in CF compared to controls and that the qualitative descriptors “chest tightness” and “inspiratory difficulty” were selected more often by CF patients compared to controls at exhaustion. In contrast, a single fatigue score, for instance captured by the ROF scale, provides only information on the severity of the symptom experienced during exercise at a given intensity. Such scales capturing perceived fatigability are however unable to shed light on the attributes of performance fatigability, i.e., the peripheral and central factors which modulate the level of fatigue experienced during a physical exercise. Peripheral muscle dysfunction and cerebral abnormalities are commons features in patients with CRD (Maltais et al., 2014; Oliveira et al., 2014; Gruet et al., 2017). Such abnormalities may be exacerbated during a physical task and contribute to exercise intolerance in these patients. Studies on performance fatigability are thus mandatory to clarify the relative role of central and peripheral mechanisms underlying increased fatigability during physical exercise in CRD.

## Performance fatigability: translation to performance and rehabilitation

### Fatigability and performance: theoretical concepts and applications

A myriad of protocols have been used over the past thirty years to investigate performance fatigability in CRD. As detailed in the next part, each protocol may have its own advantages and limits and may justify in part such degree of diversity. However, this has also lead to major impediments in understanding the causes of fatigue in CRD and translating the mechanistic research knowledge into the clinical practice. Mechanistic studies on fatigue, because they are not designed for that purpose, lack of both external and ecological validity. It is often uncertain whether the findings pertaining to a specific fatiguing task in a given CRD can be generalized to another task in patients with different phenotype severity. Another important issue is to determine whether the same laboratory findings may predict the performance of functional tasks in the real-life.

In an attempt to resolve these issues, Enoka and Duchateau (2016) recently proposed the following three-level experimental strategy (with examples of application in athletes and patients with multiple sclerosis): (1) identify a test and its main outcome of performance as reflective as possible of patients' daily activities (e.g., walking test); (2) identify a laboratory fatigability test which can strongly predict the aforementioned main outcome and (3) perform a mechanistic study to determine how a given factor (e.g., central muscle activation) underlying perceived/performance fatigability may limit performance in the laboratory test. Such approach is clearly attractive as it directly aims at translating complex mechanistic fatigue findings to the real-world performance, reducing the gap between research and clinical practice. Ultimately, a better understanding of the factors underlying reduced real-world performance will help to develop specific countermeasures.

Admittedly, the application of such model in CRD is far from being straightforward. First, it implies to identify a relevant test reflecting daily physical activities which can be influenced by performance fatigability. Both cycling and walking tests are strong indicators of health status in CRD patients. For instance, outcomes of maximal graded cycling test (e.g., peak oxygen uptake) and walking tests (e.g., 6MWT) have been repeatedly associated with quality of life and risk of death in a wide range of CRD (Nixon et al., 1992; Cote et al., 2007; Lee et al., 2009; Martin et al., 2013; Polkey et al., 2013; Hsieh et al., 2017; Layton et al., 2017). However, contrary to walking, cycling cannot be considered as a daily activity for most CRD patients. This activity notably involves a specific recruitment of leg muscles which cannot be extrapolated to daily walking.

Marquis et al. (2009) found a progressive reduction in electromyographic median frequency recorded from the *vastus lateralis* and *rectus femoris* during 6MWT in patients with severe COPD. They attributed this shift toward low frequencies as a progressive development of lower-limb fatigue during walking. However, it should be pointed out that the physiological load imposed by the 6MWT significantly varies from one patient to another, in part because of its self-paced nature. This test is thus submaximal in many CRD patients. The walk distance can even be normal in young patients with mild lung disease (e.g., patients with mild CF) and thus do not reflect the disabilities that can experience some patients during daily living. Most CRD patients choose their walking speed (i.e., preferred walking speed) in order to keep a tolerable sensation of dyspnea (Sanseverino et al., 2018) but still often present symptom of breathlessness in excess of symptoms of muscle effort. It is also important to note that walking requires the activations of many muscles groups (e.g., knee flexors and extensors, hip and ankle extensors; Franz and Kram, 2012). Considering these elements, it is unlikely that submaximal walking performance, at least on flat ground in mild to moderate CRD patients, has a significant dependence upon performance fatigability involving a specific muscle group (e.g., quadriceps). However, walking is not limited to self-paced slow speed on a flat ground and much higher muscle recruitment is required with increasing speeds and grade. This may be the case for instance during stair-climbing which is very common in and beyond the home. Dreher et al. (2008) demonstrated that physiological changes during 6MWT were not related to those during stair-climbing in severe COPD. For instance, stair-climbing resulted in higher blood lactate production (Dreher et al., 2008). This may reflect in part differences in muscle recruitment between walking on level ground and walking up, the latter likely being associated with increased muscle susceptibility to fatigue. Treadmill exercise testing with increasing grades such as the Bruce protocols or modified versions, commonly used worldwide in a variety of CRD (Klijn et al., 2003; Przybylowski et al., 2007; Cooper et al., 2010; Hebestreit et al., 2015) may thus be an ideal alternative as a reflect of real-world performance.

To date, no tests of performance fatigability have been identified as strong predictors of walking endurance in CRD. In contrast, some studies performed a fatigability test and tried to establish an a posteriori correlation with a measure of whole-body exercise capacity. For instance, Gruet et al. (2016a) found a significant association between local quadriceps endurance and peak oxygen uptake determined during graded cycling exercise in adults with CF. However, such approach is based on single and/or multiple regression analyses which are particularly sensitive to sample size and may thus suffer from lack of power. Indeed, it is often complicated to conduct large cohort studies when using complex and time-consuming mechanistic laboratory studies on fatigue. On a short-term basis, future large studies should investigate the association between one or more well-accepted measures of daily functioning and a laboratory fatigability test. This latter should be, at this preliminary stage, performed in its simplest form to increase feasibility and power sample (i.e., without the addition of complex physiological measures (e.g., EEG, neurostimulation techniques) or cognitive manipulations (e.g., addition of a concomitant cognitive task, see section Future Directions for Mechanistic Studies). Beyond walking or cycling tests, it would be also important to consider the 1-min sit-to-stand-test as an easily implementable measure of daily functional capacity in CRD. Indeed, this test reflects a movement frequently performed in daily life and has recently received increasing attention in CRD (Gruet et al., 2016b; Radtke et al., 2016; Reychler et al., 2017) with a specific multicenter validation in COPD (Crook et al., 2017), making it an interesting correlate of real-world performance. The next key question is now to identify the ideal performance fatigability test which should ideally serve both research and clinical purposes.

### Performance fatigability: which test with which fundamental characteristics?

The differences between protocols to evaluate performance fatigability in CRD rely on various factors, including the type of muscle contraction / exercise (i.e., isometric vs. isokinetic vs. whole-body exercise), the intrinsic nature of the task (i.e., based on relative vs. absolute force), its continuity (i.e., sustained vs. intermittent contractions), its intensity (i.e., maximal vs. submaximal) and the stopping criteria (i.e., fixed number of contractions vs. exhaustion). These characteristics will directly condition the possibility to gather four essential features that should demonstrate a performance fatigability test to be suitable in clinical and research settings:

1- Is the test ecologically valid?
2- Is the test reliable?
3- Is the test feasible?
4- Does this test allow easy implementation of measures of some attributes of fatigue?

#### Whole-body exercises

Performance fatigability can be evaluated from whole-body exercises which are easier to translate to real-world settings. The standard technique is to assess neuromuscular function [e.g., maximal voluntary contraction (MVC), voluntary activation, and contractile function] before and after high-intensity cycling exercise. Such studies have provided crucial insights about the relative influence of each physiological system in limiting maximal exercise capacity in CRD. They notably confirmed that, despite their ventilatory limitations, most CRD patients develop post-exercise contractile fatigue of the quadriceps, highlighting the importance of lower-limb function in the exercise intolerance experienced by these patients (Saey et al., 2005; Vallier et al., 2011; Bachasson et al., 2013c). Such methodology has also proven efficacy to detect negative response to exercise training (i.e., the absence of post-exercise contractile fatigue can mean that the exercise stimulus is not adapted to generate positive physiological adaptations, e.g., Burtin et al. (2012); see section Performance Fatigability as an Index to Detect Responders to Exercise Training). However, this whole-body approach implies that fatigability can only be assessed from pre to post maximal exercise measurements, leading to several limits. First, the degree of fatigue is largely dependent on patients' cooperation and several extra physiological factors may limit the attainment of a true maximal exercise, influencing the total amount of fatigue. Of importance, fatigue usually develops progressively during daily activities even in the absence of a maximal effort with major cardiovascular demand. Hence, measuring fatigue at a single time point (i.e., at exhaustion) may not reflect the usual level of fatigue faced by the patient. Moreover, it has been established that both peripheral and central mechanisms of fatigue recovered quickly after short-duration exercises, in healthy subjects (Froyd et al., 2013; Gruet et al., 2014) as well as in CRD patients (Gruet et al., 2016a). Thus, the time between exercise termination and post-exercise fatigue measurements should be reduced as much as possible to appreciate the full magnitude of exercise-related fatigue. Unfortunately, transferring the subjects from the ergocycle to the chair and start the neuromuscular evaluation necessitates several minutes (i.e., usually ranging from 5 to 10 min).

In an attempt to resolve this issue, Doyle-Baker et al. (2017) recently developed a new ergometer permitting to switch from recumbent cycling to isometric set-up (i.e., to measure neuromuscular function) within 1-s. This allows the measurement of neuromuscular fatigue at any moment during a cycling protocol without any time delay, providing important insights about how fatigue progressively develops during exercise for a given metabolic intensity. Such innovative ergometer may allow in the future a better understanding of the etiology of fatigue experienced by CRD patients during whole-body exercise. However, as things stand at present, such prototype is far from being included in the routine practice and the feasibility should be evaluated in CRD patients. Of importance, the ergometer has been validated with settings (articular angles) allowing electrical stimulation of the femoral nerve to assess neuromuscular function. As such method is typically poorly tolerated in fragile patients (see Section Fatiguers vs. Non-fatiguers: Methodological Concerns), slight adjustments will be necessary to allow the use of magnetic stimulation (e.g., open hip angle).

#### Local exercises

The other popular method to assess performance fatigability is to use local endurance tests involving a specific muscle group. They will be referred as “muscle fatigability” tests in the following sections. These protocols generate minimal cardiorespiratory constraint, permitting to assess muscle fatigability in isolation with oxygen delivery remaining within normal limits, as it is the case in many everyday activities. The most common maneuvers used to assess muscle fatigability are isometric and isokinetic contractions.

The isokinetic protocols used in CRD usually require the performance of repeated MVC at a given angular velocity (from 60 to 300°/s; see Evans et al. (2015b) for review). For instance Ribeiro et al. (2015) reported strong reliability of main outcomes of a quadriceps isokinetic test consisting in 30 MVC at 90°/s in moderate to severe COPD. These outcomes included total isokinetic work, peak torque and fatigue index (i.e., work performed during the last 10 repetitions/work performed during the first 10 repetitions). Such protocols may however display several disadvantages. First, the performance of repeated MVC can be largely influenced by motivational factors and osteoarticular limitations, the latter being a growing concern in the aging CRD population (Liao and Lu, 2016). This test also needs a familiarization session, costly equipment, large space requirements and trained technician, impeding its regular use in clinical practice.

Isometric protocols have also been largely utilized in CRD over the last 30 years. One of the main advantage is the low cost of an isometric set-up (i.e., custom chair and strain gauge), as compared to computerized dynamometry (i.e., at least 10 times cheaper). Strain gauge measures of quadriceps force are valid and reliable, in both healthy and CRD populations (Bachasson et al., 2013b; Machado Rodrigues et al., 2017). Isometric test have often been criticized on the basis that this muscular contraction regime is not the best reflect of daily activities of the patients. In fact, isometric contractions are often maintained for a prolonged period of time in various activities requiring the action of upper or lower limbs (e.g., holding an object, postural control). Moreover, the argument of low ecological validity could just as well work for contractions performed at constant angular velocity (i.e., isokinetic) which are non-physiological maneuvers. Isometric contractions are usually performed at a relative intensity (i.e., most often based on a given percentage of MVC) so that reduced strength of the patients will not directly influence muscle fatigability. Such methodological precaution is necessary to ensure that a specific neurophysiological abnormality related to fatigue does not only reflect a low muscle mass. This is essential when studying CRD patients as limb muscle atrophy is a common feature of these patients (Maltais et al., 2014; Gruet et al., 2017). Some studies used sustained isometric contractions until exhaustion, with a target force level usually ranging from 50 to 80% MVC (Zattara-Hartmann et al., 1995; Allaire et al., 2004; Gruet et al., 2010b; Ju and Chen, 2014; Miranda et al., 2014). The performance on such high-intensity, non-gradual protocols is in part dependent on subjects' motivation and ability to tolerate pain and thus is typically less reliable in fragile patients compared to healthy controls (Gruet et al., 2010b). Similar to whole-body exercise or continuous isokinetic protocols, sustained contractions without breaks also have the major drawback of not allowing repetitive neuromuscular evaluation throughout the task, impeding the possibility to describe the kinetics of peripheral and central mechanisms of fatigue.

A growing number of mechanistic neurophysiological investigations seek to identify the central and peripheral mechanisms contributing to increased fatigability in healthy subjects and in various chronic diseases (see Enoka and Duchateau, 2016; Twomey et al., 2017 for recent reviews). There is now accumulating evidences of brain abnormalities in CRD patients (Macey et al., 2008; Canessa et al., 2011; Dodd et al., 2012; Esser et al., 2016), notably in cortical areas implicated in motor control. Some studies in OSA and COPD demonstrated abnormalities in the premotor and primary motor cortex (e.g., altered excitability) at rest and during the performance of voluntary muscle contractions (Grippo et al., 2005; Alexandre et al., 2014, 2016). One may expect that decreased muscle performance in CRD is related in part to exaggerated and/or early exercise-induced central fatigue (i.e., abnormalities located at the spinal and/or supraspinal levels). Such hypothesis can be tested by using intermittent contractions interspaced by regular neuromuscular evaluations that may include measures of motor cortical voluntary activation and intracortical inhibitory networks. Some studies using intermittent contractions of the quadriceps have provided indirect arguments in favor of this hypothesis by showing an increased contribution of supraspinal mechanisms in fatigue-related force loss when healthy subjects faced increasing severities of acute hypoxia (Goodall et al., 2010; Rupp et al., 2015). It is only very recently that studies sought to determine whether the cortical abnormalities observed at rest in CRD patients could contribute to increased muscle fatigability and reduced endurance performance. Marillier et al. (2018a) evaluated corticospinal responses to fatiguing intermittent quadriceps contractions in patients with severe OSA and matched healthy controls. The task consisted in 17 intermittent isometric knee extensions (5-s contraction/4-s relaxation) at 35% MVC interspaced by neuromuscular evaluations (duration ~40 s) including cortical voluntary activation and intracortical inhibition assessments using single and paired transcranial magnetic stimulations (TMS). Target force was increased by 5% every two sets of contractions until task failure. Endurance time was lower in OSA patients. This was associated with lower MVC and cortical voluntary activation as well as increased silent period and long-interval inhibition throughout the fatiguing task. However, short-interval intracortical inhibition kinetics was similar between OSA and controls. Thus, exaggerated muscle fatigability in OSA can be explained in part by increased intracortical inhibitory activity of GABA_B_ receptors which can contribute to reduced voluntary activation from the motor cortex. It is of note that such cortical adjustments in OSA were present from the beginning of the task and persisted until exhaustion. This means that cortical voluntary activation deficit may be present in these patients even without a high amount of muscle fatigue.

This example illustrates the potential of intermittent isometric protocols interspaced with neuromuscular evaluations to provide important mechanistic insights into how and why fatigue develops during exercise in CRD. Further investigations should extend this paradigm to other CRD. It is also worth noting that the use of isometric contractions may facilitate the investigation of brain adaptations with fatigue by minimizing body and head movement (as compared for instance to whole-body exercises) which is essential for reducing artifacts in the data. The next issue is to determine whether an intermittent isometric test, beyond having the first attribute (i.e., easy implementation of mechanistic measurements) may also combine the three other mentioned earlier (i.e., ecological validity, reliability and feasibility) to be included in routine patients' assessments.

Bachasson et al. (2013b) developed a quadriceps intermittent fatigue (QIF) test. This test consisted in intermittent isometric knee extensions (5-s contraction/5-s relaxation) beginning at 10% MVC with a 10%-MVC increment every 10 contractions until task failure. Visual (i.e., for maintaining a target force level) and audio (i.e., soundtrack indicating the contraction-relaxation rhythm) feedbacks are provided throughout the test. Central and peripheral contributors of muscle fatigability are assessed between each set and at task failure with single and paired femoral magnetic nerve stimulations. Main outcomes are the total number of contractions performed (i.e., relative endurance index), the total force-time product (i.e., absolute endurance index) and changes in MVC (i.e., muscle fatigability index), voluntary activation (i.e., twitch interpolation technique; index of central function), M-wave, twitch and doublets at 10 and 100 Hz (i.e., indices of peripheral function). The QIF test cumulates several advantages. First, by permitting muscle reperfusion during relaxation periods (i.e., 5-s off phases), such intermittent contractions may better reflect usual muscle functioning as opposed to sustained isometric contraction which leads to muscle ischaemia. Moreover, by using progressive loading (instead of a constant-load), multiple assessments (instead of only pre vs. task failure assessments), and non-volitional contractions (i.e., induced by magnetic stimulation which induces less discomfort than electrical stimulation), this test limits the influence of pain and motivation confounding factors. The evaluation focuses on a large muscle group (i.e., quadriceps) which plays a major role in various locomotor tasks (e.g., walking, cycling). Moreover, some fatigue (i.e., reduction in twitch at set 50%MVC) and endurance (i.e., total force-time product) indices measured during this test have been significantly correlated with peak oxygen uptake measured during cycling cardiopulmonary exercise test (Bachasson et al., 2013a; Gruet et al., 2016a). The QIF test has demonstrated excellent feasibility in healthy subjects (i.e., male and female, young and older, sedentary and athletes) (Bachasson et al., 2013b, 2016) but also in various chronic conditions including fibromyalgia (Bachasson et al., 2013a), fascioscapulohumeral dystrophy, Charcot-Marie-Tooth disease (Bachasson et al., 2014) and CF (Gruet et al., 2016a), with no adverse effects. The aforementioned outcomes of the QIF test demonstrated high absolute and relative test-retest reliability with typical error expressed as a coefficient of variation and ICC ranges of 4–7% and 0.81–0.90, respectively (Bachasson et al., 2013b).

In summary, an intermittent fatiguing isometric test such as the QIF test should offer a good compromise between ecological validity, reliability and feasibility. In its current form, this test is virtually suitable for most patients with CRD and could be widely spread out in clinical settings. As mentioned earlier, the next important steps will be to determine how measures of performance fatigability as determined by this test may predict the performance of functional tasks which are good correlate of real-world performance (e.g., walking tests, 1-min sit-to-stand test, see section Fatigability and Performance: Theoretical Concepts and Applications) and then, to conduct mechanistic studies aiming to elucidate the neurophysiological underpinnings of altered performance fatigability in CRD and its interactions with perceived fatigability. The next section provides some directions for conducting such research.

#### Future directions for mechanistic studies

In its current form, the QIF test is performed using voluntary contractions and contrations evoked by femoral magnetic nerve stimulations. Peripheral nerve stimulation can distinguish for peripheral vs. central adaptations during exercise. However, this technique does not allow appraising the adaptations that occur at the cortical level with fatigue. As mentioned above, many CRD patients may present various brain abnormalities in cortical areas involved in motor control, making important the exploration of cortical functioning during exercise. Further investigations should examine the role of supraspinal mechanisms in fatigue-related force loss in CRD by coupling peripheral nerve stimulation with TMS over the motor cortex (e.g., Gruet et al., 2013; Marillier et al., 2018a). However, it is well-acknowledged that several mechanisms upstream from the motor cortex influence the execution of the motor command (see Tanaka and Watanabe (2012) for review). Different brain areas exchange information and synchronize their activities during exercise (Hilty et al., 2011; Ushiyama et al., 2011) and their complex interactions influence motor cortical functioning. It would therefore be a significant step to evaluate, besides the motor cortex, the role of other brain structures such as the premotor, the primary somatosensory and the prefrontal cortex (PFC) (e.g., Marillier et al., 2018b) in perpetuating fatigue during exercise in CRD patients. This could be achieved by augmenting neurostimulation techniques (e.g., TMS) with neuroimaging (e.g., multichannel functional near-infrared spectroscopy) and corticomuscular coherence (EEG-EMG coupling) methods. These non-invasive techniques all demonstrated good feasibility during isometric contractions in both healthy and pathological conditions and can detect small changes in brain activity with fatigue (Ushiyama et al., 2011; Gwin and Ferris, 2012; Abeln et al., 2013; Perrey, 2013; Alexandre et al., 2014; Cremoux et al., 2017; Marillier et al., 2018a). Such techniques could thus be implemented in the future during local exercises such as the QIF test, in various CRD populations.

Virtually every study investigating performance fatigability in CRD used single motor tasks. In these laboratory situations, the influence of perceived fatigability on performance fatigability is limited, notably because of the absence of any specific cognitive stress. Another possibility is thus to investigate performance fatigability with the addition of a concomitant cognitive task. Cognitively demanding motor tasks are relevant to functional activities in daily-life and investigating performance fatigability in such conditions would thus increase ecological validity of the findings. In healthy subjects, the addition of a concomitant cognitive task to a motor task impaired motor performance, notably by reducing the time to task failure in the dual-task compared to the motor task performed alone, implying increased performance fatigability (Yoon et al., 2009; Mehta and Agnew, 2011; Keller-Ross et al., 2014). It is proposed that this effect could be even more pronounced in CRD and cognitive-motor dual tasks may represent situations particularly prone to induce exaggerated fatigability in CRD patients. Cognitive impairments are frequent in CRD, with higher prevalence compared to the general population. For instance, high level of cognitive failure was found in 35% of the patients with sarcoidosis and only in 14% of the age- and sex-matched healthy controls (Elfferich et al., 2010). Fairly similar prevalence was found in COPD (Villeneuve et al., 2012; Torres-Sanchez et al., 2015). These cognitive deficits, and notably the loss of executive functions (Andrianopoulos et al., 2017), will make more difficult to maintain the performance of both cognitive and motor tasks and will require greater brain resources to execute them simultaneously. Such assumptions are supported by recent findings in old healthy subjects (assumed to be more prone to cognitive abnormalities), who demonstrated reduced endurance during a motor task (i.e., handgrip at 30% MVC until exhaustion) as compared to young healthy subjects, but only when this motor task was performed with a concomitant cognitive task (i.e., mental arithmetic) (Shortz and Mehta, 2017). Previous studies demonstrated that some brain regions, such as the PFC, play an important role in regulating performance during cognitive-motor dual tasks. Reduction in PFC activity has been associated with reduced time to task failure during exercises with elevated cognitive demands (Mehta and Parasuraman, 2014; Shortz et al., 2015). This effect can be more pronounced in CRD patients as they need greater mobilization of brain resources to execute the same task. Blunted PFC activity and even disengagement may occur during cognitive-motor dual tasks in CRD, affecting the functioning of other interconnected brain regions such as the motor cortex. It can be thus speculated that alterations in performance fatigability in CRD would be even more marked during cognitive-motor dual tasks due to early central abnormalities impacting the ability to sustain a high level of voluntary activation. Moreover, the addition of a cognitive task (that is harder to perform for CRD patients) may exacerbate the influence of most factors of the psychosocial domain of perceived fatigability (see Figure 1). For instance, reduced motivation, negative mood, decreased executive functions and negative performance feedback may all, alone or in combination, contribute to increased muscle fatigability through earlier disengagement from the motor task. These assumptions could be tested by implementing concomitant cognitive tasks and assessments of perceived fatigability throughout the QIF test. For instance, mental arithmetic (e.g., subtraction from a 4-digit number by 13; Yoon et al., 2009) or memory tasks (e.g., memorize a sequence of numbers) could be superimposed during the 5-s contraction period with the answer expected during the 5-s recovery period. Perceived fatigability could be assessed using the ROF scale every 10 contractions. Motor cortex and PFC activations could be assessed by TMS and functional near-infrared spectroscopy, respectively.

It is also worth noting that cognitive failure has recently been demonstrated as an important predictor of elevated perceived fatigability (evaluated by the Fatigue Assessment Scale at baseline and then at 6 and 12 months) in patients with sarcoidosis (Hendriks et al., 2018). Thus, it is proposed that cognitive impairments may contribute to both the trait and the state level of fatigue in CRD. Cognitive-motor dual tasks may also serve rehabilitation purposes as producing more fatigue during a given session may further trigger positive physiological adaptations in CRD (see section Fatiguers as Responders: Experimental Evidences and Exercise Training Strategies). Mechanistic studies aiming to clarify the neurophysiological adaptations to cognitively demanding motor tasks in CRD may thus help to design future interventions for these patients.

### Performance fatigability as an index to detect responders to exercise training

#### Fatiguers as responders: experimental evidences and exercise training strategies

Exercise training has already proven several beneficial effects in various CRD (McCarthy et al., 2015; Aiello et al., 2016; Radtke et al., 2017) and is an integral part of the package of care offered to most patients. However, there is still large inter-individual variability regarding its effectiveness, especially for cardiorespiratory fitness, and some CRD patients receive only few or no benefit at all from exercise training. This has led to the so-called concept of “responders” and “non-responders” to exercise training, in both healthy (e.g., Mann et al., 2014) and pathological conditions (e.g., CRD, Troosters et al., 2001). Several studies tried to elucidate the factors associated with inter-individual differences in response to standardized exercise training. Much attention has been directed toward the role of genetic factors (Bouchard et al., 2011; Bouchard, 2012; Sarzynski et al., 2017). In their HERITAGE Family Study, Bouchard et al. (2011) found that 21 single-nucleotide polymorphisms explained 49% of the variance in maximal oxygen uptake trainability following a 20-week exercise training program in 473 sedentary adults. Some reports involving CRD patients also suggest an influence of genetic determinants in trainability. For instance, Jarosch et al. (2016) found an increase in the oxidative myofibre type I proportion in COPD patients with PiMM genotype but not in those with PiZZ genotype following a 3-week exercise training program, suggesting a better trainability for the PiMM genotype. However, this study was small and cardiorespiratory fitness was not evaluated. Moreover, it is still unknown whether the outcomes from the large HERITAGE study can be extrapolated to CRD patients. It is likely that hereditary influences the pre-training (i.e., baseline) phenotype buy has only a minor influence on the subsequent training response (see Mann et al., 2014 for review). Individual variation in trainability that cannot be explained by hereditary can thus probably be related to the characteristics of the training program.

Montero and Lundby (2017) recently challenged the notion of non-response to exercise training in healthy adults. They allocated 78 subjects into five groups that performed one, two, three, four and five 1-h endurance training sessions per week, for a 6-week period. Non-response to the intervention was defined as a change in maximal incremental exercise power output within the typical error of measurement (i.e., ±3.96%). Non-responders participants were then enrolled in another 6-week endurance training period which included two additional sessions per week. The main result is that after the second period of training, the non-response was abolished in all individuals. This means that the non-response to exercise training is mainly dependant on the dose of exercise, and then increasing the overall load (≥240 min per week in the study by Montero and Lundby, 2017) should be sufficient to trigger positive adaptations. Even though such findings are robust in healthy individuals, it is currently unknown whether they can be extrapolated to CRD patients. First, it may be complicated to increase the overall dose of training in some patients groups due to logistical issues (e.g., time spent to treatments, see also section Fatigue as a State: Time for Ecological Momentary Assessment), especially in the hospital setting. Moreover, several psychosocial and physiological factors may impede the patients to reach training intensities which are compatible with positive adaptations. Thus, even with an assumed optimal overall training dose, the exercise program may not be effective due to inadequate training characteristics (e.g., duration, intensity) at the level of a given training session. As acknowledged by Montero and Lundby (2017), their study was designed to investigate the independent contribution of overall training dose but not its components, at a given exercise session level, which may exert an important influence in CRD patients. This is particularly true when considering the likely phenotypic heterogeneity in a given group of CRD patients involved in a pulmonary rehabilitation program (e.g., patients with/without limb muscle weakness, with/without substantial ventilatory limitation (e.g., Troosters et al., 2001). The large heterogeneity in factors limiting exercise tolerance in CRD patients and the resulting individual variations in homeostatic stress induced by a training session clearly warrants a careful individualized approach. The next fundamental question is now how to determine whether a given session is effective for a given CRD patient?

Following the well-known principle of muscle loading, production of acute muscle fatigue (i.e., increase in performance fatigability) can be viewed as a positive response to a given exercise training session. Conversely, the absence of exercise induced-muscle fatigue implies that the stimulus may not be sufficient to trigger positive physiological adaptations (e.g., structural adaptations within the muscle fibers). Within this theoretical framework Burtin et al. (2012) thought to determine whether the muscle fatigue exhibited by patients with COPD after an acute exercise session could be used as an indicator to detect the future responders to a whole exercise program. Forty six patients with COPD completed a 3-month multimodal exercise program. Exercise capacity (e.g., 6MWT, peak workload during graded cycling exercise test), quadriceps strength and quality of life were assessed before and after the program. The training consisted in various exercise modalities including cycling, treadmill walking, stair climbing and quadriceps resistance exercise. Muscle fatigue was evaluated after 1 month of training following a single exercise session. Significant contractile fatigue was defined as a drop in resting quadriceps potentiated twitch (elicited by femoral nerve magnetic stimulation) ≥15% after the exercise session. Twenty-nine patients (63%) developed contractile fatigue after exercise according to this criterion. This subgroup of patients demonstrated greater improvements in both exercise capacity and quality of life after the 3-month program, as compared to patients who did not exhibit post-exercise fatigue. These results will be subsequently confirmed in a larger cohort (*n* = 132), with patients capable to develop quadriceps contractile fatigue having greater improvements in 6-min walk distance after exercise training (Mador et al., 2014). In practice, identifying non-fatiguers patients may prevent to enroll them in a long-term ineffective exercise program. Measurement of post-exercise muscle fatigue is clearly a promising technique in that respect and future studies should extend the use of such methodology to other CRD.

The fundamental issue is now to understand why a substantial percentage of patients are not able to develop significant muscle fatigue following an exercise session. First, following the dose-dependent principle of Montero and Lundby (2017) and applying it at the session level, it is still possible that the intensity and/or duration of the session is not optimal. However, in the study by Burtin et al. (2012), training intensity was high and not different between the fatiguers and non-fatiguers groups. Some patients may not develop muscle fatigue of the active limbs because major ventilatory constraints impede the achievement of a sufficient training stimulus. Such hypothesis is partially supported by findings showing that COPD patients exhibiting less ventilatory limitation to acute exercise are more prone to improve following a whole exercise program (Troosters et al., 2001). However, the percentage of variability explained by this factor is limited and there was no evidence of different patterns of ventilatory limitation during exercise between fatiguers and non-fatiguers in the study by Burtin et al. (2012). Additional factors may thus limit the susceptibility to develop muscle fatigue. These may include intrinsic muscles adaptations such as poor skeletal muscle glycolytic enzyme activity (Saey et al., 2005). However, beyond physiological factors, it is also possible that psychosocial factors may alter the ability of some patients to produce muscle fatigue, notably through poor motivation to push themselves during each session of the program. Some patients may present several barriers to physical exercise linked to self-efficacy beliefs and symptom severity. In particular, negative outcome expectations (e.g., increased perceived fatigability, worsening symptoms, dyspnea-related fear) and disregard of the potential benefits of exercise training (e.g., lack of a clear positive effect on resting lung function), associated with the negative feeling of being too old to exercise (e.g., COPD patients, many OSA patients) (Kosteli et al., 2017) may all impact immediate engagement in a given exercise session. Importantly, such negative beliefs can be associated over time with feeling of weariness due to lack of exercise diversity. This can affect long-term engagement and thus the ability to produce repeated fatigue from one session to another over the whole program duration.

**Figure 2:**
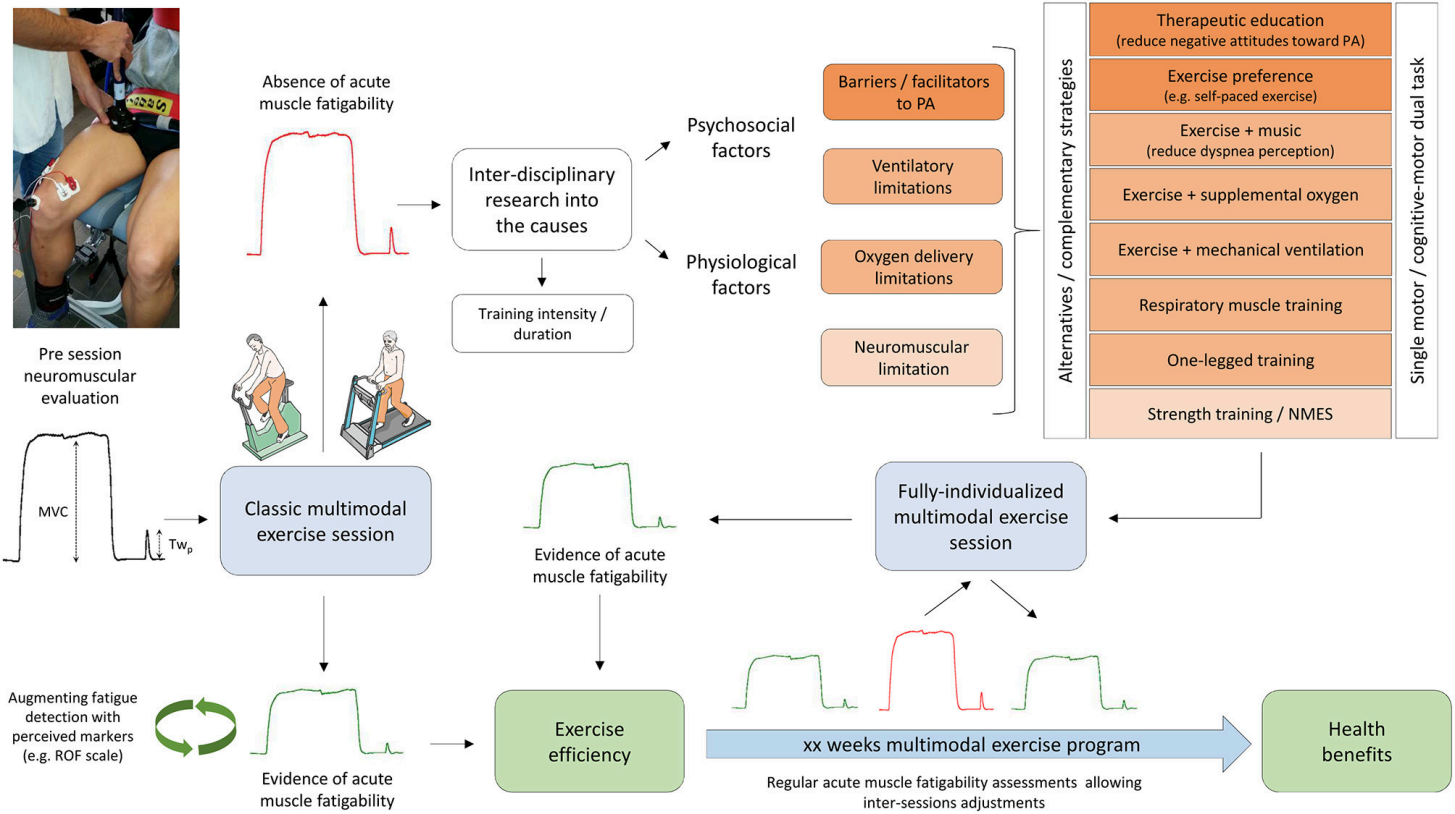
An important clinical application of the measurement of performance fatigability is to evaluate the acute muscle fatigability following an exercise rehabilitation session as a surrogate of efficient muscle loading. Such technique may help identifying future responders and non-responders to an exercise program of several weeks/months. Patients who exhibit fatigue [e.g., reduction in the amplitude of the potentiated twitch (Tw_p_) elicited by femoral magnetic nerve stimulation] following an acute exercise session can be classified as responders to the intervention and are more likely to develop positive adaptations following the whole program than patients who do not exhibit fatigue. For these latter, the next step is to identify the factors preventing the development of acute muscle fatigue on an individual basis. The proposed inter-disciplinary approach should permit to evaluate the relative influence of physiological, psychological and sociological factors. Various alternatives strategies can be offered to trigger optimal muscle loading. The effectiveness of some of these strategies to produce acute muscle fatigue remains to be confirmed, as well as the potential additional benefits of incorporating a concomitant cognitive component to these strategies. The current list is thus proposed as an initial framework that should be modified and/or completed according to future experimental results. A given exercise strategy may be effective over a short time period. However, it may become less effective in the long term. Thus, regular acute muscle fatigability assessments should be performed with the aim to adjust training modalities if necessary and then promote long-term adherence and health benefits. Treadmill and cycling exercise illustrations were modified from Smart Servier Medical Art on April 2018, available online at https://smart.servier.com/category/general-items/equipment/, licensed under the CC BY 3.0 license. MVC, maximal voluntary contraction; NMES, neuromuscular electrical stimulation; PA, physical activity; ROF, rating-of-fatigue.

In view of the multitude of factors potentially contributing to the absence of exercise-induced muscle fatigue, it seems essential to promote an inter-disciplinary approach assessing the relative influence of physiological, psychological and sociological factors in impeding muscle susceptibility to fatigue. Various alternatives to classic exercise rehabilitation sessions can thus be proposed and, by limiting the negative influence of a given factor or symptom, they should theoretically lead to optimal muscle loading. Such strategies (Figure 2) may directly aim at:

1- Modulating patients' personal barriers and enablers to exercise training (e.g., therapeutic education) and motivation (e.g., use of self-paced exercise to increase patients' autonomy and long-term adherence to exercise through the promotion of a more intrinsically motivated exercise behavior). The use of specific exercise preference inventory (e.g., such as the SEPI questionnaire by Bonner et al., 2016, specifically validated in stroke, but with items easily adjustable to CRD patients) may also guide the choice of specific exercise modalities.
2- Acting against the negatives feelings associated to dyspnea through desensitization techniques (e.g., listening self-selected music, Lee et al., 2018);
3- Reducing the demand imposed on the cardiopulmonary system allowing to reach higher training intensities that would not be normally possible for patients with important ventilatory limitation. They may include exercise with supplemental oxygen (Emtner et al., 2003) or non-invasive ventilation (Ambrosino and Xie, 2017), one-legged cycling (Evans et al., 2015a) or combination of aerobic training with respiratory muscle training (Santana-Sosa et al., 2014).
4- Increasing limb muscles mass and force, especially in very deconditioned patients to facilitate the completion of a subsequent whole-body exercise program. For instance, 6 weeks of neuromuscular electrical stimulation training conducted prior 8 weeks of endurance training in severe patients with CF was effective to increase quadriceps muscle force which in turn was related to decreased ventilatory requirement during exercise (Vivodtzev et al., 2013). The ability of some of these strategies to induce significant acute muscle fatigue remains to be confirmed.

The addition of concomitant cognitive tasks to these strategies may also be a promising lead for the future. As proposed above, it may help to produce more fatigue for the same intervention duration (see section Whole-Body Exercises). Moreover, in addition to the playful aspect which may promote long-term adherence, cognitive-motor dual tasks may also serve to practice various cognitive aptitudes, notably executive functions which are impaired in many CRD patients (Andrianopoulos et al., 2017).

#### Fatiguers vs. non-fatiguers: methodological concerns

As discussed above, these experimental findings clearly demonstrate the relevance of measuring muscle fatigue to predict the effectiveness of exercise training in CRD. However, several methodological concerns should be addressed before thinking to implement this technique into clinical practice. The method to distinguish fatiguers from non-fatiguers is based on the fall in potentiated twitch >15% measured few minutes after the end of an exercise session. The use of a non-volitional index such as potentiated twitch for stratification is clearly relevant in patients as it should be more sensitive than a volitional index of muscle fatigue (i.e., MVC) which depends on patient's cooperation and motivation. However, the use of this unique index may also have some inconvenient. First, it implies the use of costly equipment (i.e., magnetic stimulator and coil) and trained investigators to obtain reliable measurements. Second, potentiated twitch was measured in both studies from femoral nerve stimulation. This seems logical as lower-limbs muscles are particularly affected in CRD (Maltais et al., 2014; Gruet et al., 2017) and a large component of exercise programs is designed to predominantly solicit this muscle group (e.g., cycling, walking, climbing stairs). Nevertheless, arm muscles are also affected in many CRD patients and upper-limb exercise training is also sometimes incorporated in pulmonary rehabilitation programs (McKeough et al., 2016). Thus, the use of the sole femoral potentiated twitch may not be representative of the fatigue experienced during a multimodal (i.e., involving both lower and upper limbs) exercise session. In addition, it is unknown whether the 15% threshold could be applied for other muscles groups. It is also worth noting that potentiated twitch cannot be measured for every muscle groups due to the induction of the co-contraction of the antagonist muscles, affecting the mechanical response (e.g., see Millet et al., 2011 for review). Moreover, supramaximal stimulation is necessary to ensure full spatial recruitment even with slight changes in electrode (i.e., electrical stimulation) or coil positioning (i.e., magnetic stimulation). As such, a stimulation intensity ranging from 120 to 150% of optimal intensity is usually chosen for electrical stimulation. Unfortunately, contrary to electrical stimulators, current magnetic stimulators are limited in power output and it has been demonstrated that excessive fat thickness in the femoral region could preclude the achievement of supramaximal stimulations (Tomazin et al., 2011), making this technique inoperable in some patients. This is of particular importance in chronic respiratory disorders since overweight and even obesity are important and growing concerns in various CRD, including COPD (Rutten et al., 2013), OSA (Romero-Corral et al., 2010), and even CF (Hanna and Weiner, 2015). Next generation of magnetic stimulators should address this important issue. Making this technique usable on a larger sample-scale would undoubtedly further foster its implementation into the clinical practice.

As multiple nerve assessments may be complicated for methodological and logistical reasons, it may also be of interest to add an indicator of perceived fatigability when a whole-body exercise session is intended. For instance, the ROF scale (Micklewright et al., 2017; see also section Fatigue as a State: Time for Ecological Momentary Assessment), which also demonstrates high convergent validity during post-exercise recovery, may be used in conjunction to femoral magnetic nerve stimulation to obtain further insights on the fatiguing aspect of an exercise session. Further studies are warranted to identify the usefulness and potential implementation of perceived indicators of fatigue. An important last notion is that a fatiguer, as determined from one single session, may not inevitably be a good training responder on a long-term basis. Although a given exercise modality may be effective at a single time point, it is possible, even when adjusting for the intensity according to patients' improvements, that such modality loses effectiveness over time (because of motivational factors, for instance). Thus, acute muscle fatigability assessments should be conducted on a periodic basis, allowing regular inter-sessions adjustments for optimal long-term adherence and health benefits (Figure 2).

## Summary and futures directions

Fatigue is prevalent in CRD and negatively impacts the daily lives of the patients. A better knowledge of the modulators and attributes of fatigue is thus fundamental to design appropriate countermeasures. To this end, fatigue must be regarded as a multifaceted phenomenon that should be described with an inter-disciplinary approach, giving consideration to both its perceptual and performance components. Most studies in CRD evaluated perceived fatigability as a trait characteristic using multidimensional scales. Such studies provided important insights regarding the prevalence, the etiology and the clinical impact of an elevated trait level of fatigue. However, fatigue is an unstable, dynamic phenomenon which can arise from various real-life situations with varying degrees of severity. This is particularly the case for CRD which are evolving diseases characterized by frequent events (e.g., hospitalizations, changes in medication, new long-term treatment) that may dictate the fatigue level. Evaluating fatigue as a state, at several time points in various contexts and environments is critical to improve our understanding of how fatigue affects the daily-life of CRD patients in real-world settings. Thus, traditional scales of fatigue should be supplemented with ecological assessment of fatigue. Future studies should implement valid fatigue scales (e.g., ROF scale) into mobile-EMA platforms and determine how fatigue is modulated within and between days, according to specific events (e.g., occurrence and treatment of an exacerbation). The state level of fatigue must also be considered during a physical task. This specific situation can induce substantial deviations in the attributes of perceived and performance fatigability and severe fatigue can be expected during exercise in CRD patients. Performance fatigability is determined by the neuromuscular adjustments that occur to meet the demands of the motor task. Both peripheral and central systems may exhibit abnormalities in CRD and future studies should elucidate whether neuromuscular alterations observed at rest may be exacerbated during physical exercise and could negatively impact performance in these patients. To this end, an isometric laboratory test such as the QIF test could represent an ideal compromise between feasibility, reliability and ecological validity, while being able to implemental neurophysiological and neuroimaging methods. A better understanding of the neurophysiological underpinnings of performance and perceived fatigability and their impact on real-world performance (e.g., walking performance, sit-to-stand capacity) will foster the development of new strategies mitigating the influence of the attributes of fatigue on patients' performance. Such knowledge will also serve to develop new strategies promoting the development of acute muscle fatigue, a surrogate of efficient muscle loading during exercise training sessions. Various factors may limit the ability to produce muscle fatigue during exercise training in CRD patients. They should by identified by adopting a comprehensive inter-disciplinary approach giving consideration to physiological, psychological and sociological factors that may potentially hinder the development of muscle fatigue in these patients. Future fatigue research should determine whether a given individualized exercise intervention which has proved its effectiveness in triggering acute muscle fatigue production will ultimately leads to reduced levels of perceived fatigability into the everyday lives of the patients.

## Author contributions

The author confirms being the sole contributor of this work and has approved it for publication.

### Conflict of interest statement

The author declares that the research was conducted in the absence of any commercial or financial relationships that could be construed as a potential conflict of interest.

## Abbreviations

6MWT: six-minute walk test
CF: cystic fibrosis
COPD: chronic obstructive pulmonary disease
CRD: chronic respiratory diseases
EMA: ecological momentary assessment
MVC: maximal voluntary contraction
OSA: obstructive sleep apnea
PFC: prefrontal cortex
QIF: quadriceps intermittent fatigue
ROF: rating-of-fatigue
RPE: ratings of perceived exertion.
